# Transient vitreous opacity following combined intravitreal injection of pegcetacoplan and faricimab-svoa in patients with neovascular age-related macular degeneration and geographic atrophy

**DOI:** 10.1016/j.ajoc.2026.102545

**Published:** 2026-02-12

**Authors:** Ying Zhang, Carissa Wei, Jay M. Stewart

**Affiliations:** a University of California, San Francisco, Department of Ophthalmology, San Francisco, CA, United States; b Zuckerberg San Francisco General Hospital and Trauma Center, Department of Ophthalmology, San Francisco, CA, United States; c Department of Ophthalmology, Qilu Hospital, Cheeloo College of Medicine, Shandong University, Jinan, China

**Keywords:** Geographic atrophy, Neovascular age-related macular degeneration, Vitreous opacity, Pegcetacoplan, Faricimab-svoa

## 1 Case report

### 1.1 Case 1

**Fig. 1:**
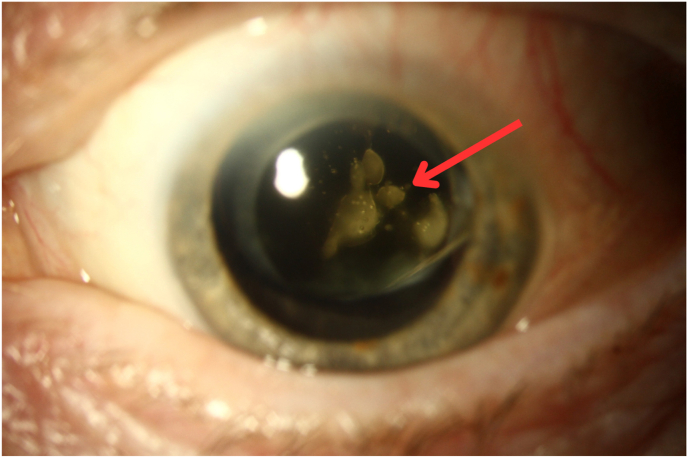
Slit lamp photograph from Case 1, demonstrating vitreous opacity (red arrow) that appeared after immediate sequential injection of pegcetacoplan and faricimab-svoa. (For interpretation of the references to color in this figure legend, the reader is referred to the Web version of this article.)

An 82-year-old woman with neovascular age-related macular degeneration (nAMD) and geographic atrophy (GA) in the left eye received simultaneous intravitreal injections of pegcetacoplan (15 mg, 0.1 mL) and faricimab-svoa (6 mg, 0.05 mL), both administered in the superotemporal quadrant at adjacent sites, using separate syringes and needles. Anterior chamber paracentesis was performed to reduce intraocular pressure (IOP) prior to injection. Immediately after the injections, she reported painless central vision loss. Best-corrected visual acuity (BCVA) declined from 20/25 to hand motions, while IOP and anterior segment findings remained normal. A large opacity was observed in the central anterior vitreous (Fig. 1). Within three hours, BCVA returned to baseline, and the opacity resolved without intervention.

### 1.2 Case 2

A 77-year-old woman with nAMD and GA in the left eye underwent the same procedure. She reported dim vision immediately post-injection. BCVA, IOP, and anterior segment findings remained unchanged. On fundus examination, a small vitreous opacity was visualized in the superotemporal quadrant, which resolved spontaneously thereafter.

**Fig. 2:**
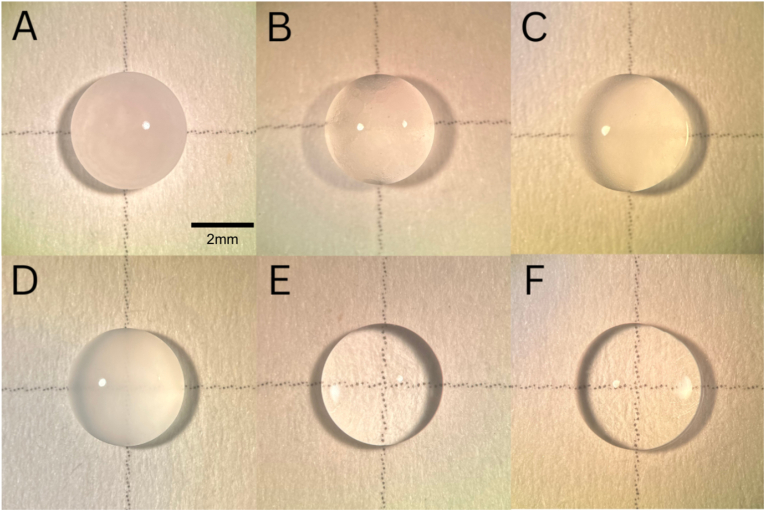
Opacification observed in the medicine mixtures compared with balanced salt solutions (BSS) control. The pegcetacoplan–faricimab-svoa combination showed the most pronounced opacification, whereas the pegcetacoplan–ranibizumab mixture showed the least. The appearance of the opacity did not change as the solutions dried. (A) faricimab-svoa and pegcetacoplan. (B) aflibercept high-dose and pegcetacoplan. (C) aflibercept and pegcetacoplan. (D) bevacizumab and pegcetacoplan. (E) ranibizumab and pegcetacoplan. (F) BSS alone.

An *in vitro* experiment was performed to assess whether a potential interaction between medications could be responsible for the observed opacity in these two cases. Pegcetacoplan was mixed with either faricimab-svoa, bevacizumab, aflibercept, aflibercept high-dose (HD), or ranibizumab at ratios described in Supplemental Table 1. Opacification of varying degrees occurred, most prominently with faricimab-svoa and least with ranibizumab. Balanced salt solution (BSS) served as a control (Fig. 2).

**Fig. 3:**
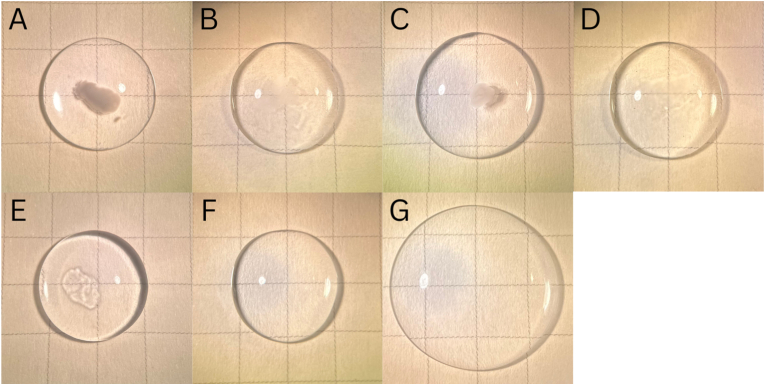
Opacification observed in medicine mixtures injected into the center of a 200 μL aliquot of porcine vitreous, visualized against grid paper (each box is 5 mm × 5 mm). (A) faricimab-svoa and pegcetacoplan. (B) aflibercept high-dose and pegcetacoplan. (C) aflibercept and pegcetacoplan. (D) bevacizumab and pegcetacoplan. (E) ranibizumab and pegcetacoplan. (F) Porcine vitreous alone. (G) The opacity resulting from the mixture of faricimab-svoa and pegcetacoplan disappeared after 225 μL of BSS was added and gently agitated.

To simulate intravitreal conditions, for each pair of co-injected medications, 5 μL of pegcetacoplan was injected into 200 μL aliquots of porcine vitreous, followed by 2.5–3.5 μL of the corresponding medication (Fig. 3A–F). The greatest opacification again occurred with faricimab-svoa. (The opacity became less dense over the course of one hour (not shown)). After adding 225 μL of BSS and gently agitating the sample to simulate eye movement, the opacity gradually dispersed, mirroring clinical observations (Fig. 3G). Both *in vitro* experiments were repeated five times and showed consistent results.

## 2 Discussion

Based on the clinical observations in these cases, a dense vitreous opacity may appear acutely after combined administration of pegcetacoplan and faricimab-svoa. Though the mechanism is unclear, our *in vitro* findings suggest a reversible formulation incompatibility between these medications, particularly with faricimab-svoa, which can resolve spontaneously, presumably through interaction with vitreous and movement that disperses the opacity. While previous studies have investigated similar opacification between vancomycin and ceftazidime delivered via different syringes and needles, interaction between pegcetacoplan and other nAMD medications represents a scenario that could be observed more frequently due to the prevalence of AMD.^1^ Vitreous opacities have also been reported following intravitreal brolucizumab injections, though these cases were observed with brolucizumab alone and not in combination with other intravitreal medications.^2^ Therefore, based on our observations, future studies should investigate the potential mechanisms behind this interaction, perhaps considering factors such as drug solution pH, ionic strength, and viscosity. In addition, we noted in the *in vitro* experiment that the mixture containing aflibercept HD appeared less opaque compared with aflibercept, for reasons that will require further investigation (Fig. 3B).

Clinicians may consider extending the time between injections or performing them on separate days to minimize risk of this vitreous opacity developing. However, as the prevalence of patients with concurrent nAMD and GA increases, administering the two injections at the same sitting, as in these instances, can help to minimize treatment burden for both the patient and the clinic. Managing the nAMD and GA separately would otherwise require additional follow-up visits, which requires additional logistical support and can be particularly burdensome for patients who live far from the clinic. If vitreous opacification occurs following co-injection, observation is recommended, as it may clear without intervention, based on the observations in these two cases.

## 3 Conclusions

Awareness of this transient complication resulting from simultaneous administration of pegcetacoplan and faricimab-svoa or other nAMD injections can prevent unnecessary diagnostic or surgical intervention. Future studies should further characterize this interaction and its clinical significance.

## CRediT authorship contribution statement

**Ying Zhang:** Writing – original draft, Methodology, Data curation. **Carissa Wei:** Writing – original draft, Resources, Project administration, Methodology, Investigation. **Jay M. Stewart:** Writing – review & editing, Supervision, Resources, Project administration, Funding acquisition, Data curation, Conceptualization.

## Patient consent

Written consent to publish this case has not been obtained. This report does not contain any personal identifying information.

## Funding

Supported in part by the 10.13039/100000053National Eye Institute (Core Grant for Vision Research, EY002162); Research to Prevent Blindness, Inc., New York, NY; All May See Foundation, Inc., San Francisco, CA; Young Elite Sponsorship Program of Shandong Provincial Medical Association, Shandong, China. The funding organizations had no role in the design or conduct of this research.

## Declaration of competing interest

The authors declare that they have no known competing financial interests or personal relationships that could have appeared to influence the work reported in this paper.
