# Supplementary material for: Transient vitreous opacity following combined intravitreal injection of pegcetacoplan and faricimab-svoa in patients with neovascular age-related macular degeneration and geographic atrophy
Source: Am J Ophthalmol Case Rep. 2026 Feb 12;42:102545. doi: 10.1016/j.ajoc.2026.102545 (PMC12934209; doi:10.1016/j.ajoc.2026.102545)
Supplement: Multimedia component 1 [file mmc1.pdf]

Supplemental Table 1. In vitro drug volumes and doses scaled from FDA-approved clinical injection parameters.

Drug Name	Volume of Drug Used (µL)	Dose of Drug Used (mg)	Volume of Pegcetaco-plan	Dose of Pegcetaco-plan	FDA-Approved Clinical Volume (µL)	FDA-approved Clinical Dose (mg)
Faricimab-svoa	2.5 µL	0.3 mg	5.0 µL	0.75 mg	0.05 mL	6 mg
Bevacizumab	2.5 µL	0.0625 mg	5.0 µL	0.75 mg	N/A*	N/A*
Aflibercept	2.5 µL	0.1 mg	5.0 µL	0.75 mg	0.05 mL	2 mg
Aflibercept HD	3.5 µL	0.4 mg	5.0 µL	0.75 mg	0.07 mL	8 mg
Ranibizumab	2.5 µL	0.025 mg	5.0 µL	0.75 mg	0.05 mL	0.5 mg
Pegcetacoplan	5.0 µL	0.75 mg	---	---	0.1 mL	15 mg

In vitro drug amounts were based on FDA-approved injection volumes described in each medication's prescribing information. Assuming the average adult vitreous volume is approximately 4 mL, we followed a ratio of 4 mL vitreous : 0.1 mL pegcetacoplan : 0.05 mL of the co-administered nAMD medication, except for aflibercept HD, which was modeled at 0.07 mL. These ratios were proportionally scaled down to the smaller experimental volumes shown above.

* Bevacizumab volume was based upon the typical clinical dose of 1.25 mg in 0.05mL[1].

Reference

1. Avery RL, Pieramici DJ, Rabena MD, Castellarin AA, Nasir MA, Giust MJ. Intravitreal bevacizumab (Avastin) for neovascular age-related macular degeneration. Ophthalmology. 2006 Mar;113(3):363-372.e5. doi:

10.1016/j.ophtha.2005.11.019. Epub 2006 Feb 3. PMID: 16458968.
